# Diagnostic Performance and Kinetics of Hepatitis E Viral RNA and IgM Antibody Test Positivity in a Genotype 1 Outbreak in South Sudan

**DOI:** 10.1093/infdis/jiaf436

**Published:** 2025-08-14

**Authors:** Aybüke Koyuncu, Robin C Nesbitt, Catia Alvarez, Kinya Vincent Asilaza, Joseph Wamala, Melat Haile, Etienne Gignoux, Manuel Albela, Emily S Gurley, Frederick Beden Loro, Duol Biem, Monica Rull, John Rumunu, Iza Ciglenecki, Isabella Eckerle, Andrew S Azman

**Affiliations:** Department of Epidemiology, Johns Hopkins University, Baltimore, Maryland, USA; Epicentre, Paris, France; Epicentre, Paris, France; Geneva Centre for Emerging Viral Diseases, University Hospitals of Geneva and University of Geneva, Geneva, Switzerland; Médecins Sans Frontières, Juba, South Sudan; World Health Organisation, Juba, South Sudan; Médecins Sans Frontières, Geneva, Switzerland; Epicentre, Paris, France; Médecins Sans Frontières, Geneva, Switzerland; Department of Epidemiology, Johns Hopkins University, Baltimore, Maryland, USA; World Health Organisation, Juba, South Sudan; South Sudan Ministry of Health, Juba, South Sudan; Médecins Sans Frontières, Geneva, Switzerland; South Sudan Ministry of Health, Juba, South Sudan; Médecins Sans Frontières, Geneva, Switzerland; Geneva Centre for Emerging Viral Diseases, University Hospitals of Geneva and University of Geneva, Geneva, Switzerland; Department of Medicine, Faculty of Medicine, University of Geneva, Geneva, Switzerland; Department of Epidemiology, Johns Hopkins University, Baltimore, Maryland, USA; Geneva Centre for Emerging Viral Diseases, University Hospitals of Geneva and University of Geneva, Geneva, Switzerland; Médecins Sans Frontières, Geneva, Switzerland; Division of Tropical and Humanitarian Medicine, University Hospitals of Geneva, Geneva, Switzerland

**Keywords:** hepatitis E, hepatitis E virus (HEV), diagnostics

## Abstract

**Background:**

Diagnostics are essential for understanding hepatitis E epidemiology, but the field performance of available tests remains unclear. We evaluated the performance of polymerase chain reactions (PCR), IgM ELISA, and the Assure hepatitis E virus (HEV) IgM rapid diagnostic test (RDT) during a HEV genotype 1 outbreak and assessed the duration of viremia and antibodies responses.

**Methods:**

We used data from enhanced surveillance at a health facility in Bentiu internally displaced persons camp, South Sudan (March-December 2022). As part of a vaccine effectiveness study, suspected hepatitis E cases underwent testing with all 3 diagnostics at enrollment with a follow-up sample. We used a latent class model to estimate test performance and accelerated failure time models to estimate time from jaundice onset to a negative test for PCR and ELISA.

**Results and Conclusions:**

Among 893 suspected cases, test sensitivity declined with time from jaundice onset. Within 30 days of jaundice onset, PCR sensitivity was 73% (95% Credible Interval [CrI] 27, 90), compared to 86% for RDT (95% CrI: 74, 93), and 95% for ELISA (95% CrI: 91, 98). Specificity was high across tests: PCR at 98% (95% CrI: 98, 99), RDT at 95% (95% CrI: 93, 96), and ELISA at 95% (95% CrI: 93, 96). Median time from jaundice onset to negative test was 19 days (95% CI: 17, 21) for PCR and 113 days (95% CI: 87, 163) for ELISA. The Assure IgM RDT showed higher sensitivity for identifying hepatitis E than PCR and similar specificity to IgM ELISA, supporting its use in surveillance. Care seeking delays can greatly influence the interpretation of diagnostic tests.

Hepatitis E causes an estimated 3 million symptomatic cases of acute viral hepatitis each year with case fatality risks of up to 25% often reported among pregnant women [1, 2], though the true burden is unknown. Large outbreaks, with thousands of cases, caused by hepatitis E virus (HEV) genotypes 1 and 2 occur due to fecal contamination of drinking water [3].

Understanding the true burden of hepatitis E is challenging due to lack of routine testing for hepatitis E among acute jaundice cases, particularly in low- and middle-income settings. A safe and effective vaccine for hepatitis E exists (Hecolin; Innovax, Xiamen, China) [4], but the World Health Organization (WHO) has identified gaps in burden of disease data as a barrier to widespread introduction of the vaccine [5]. The vaccine is currently only recommended by WHO for use in outbreak settings [5].

Many settings where hepatitis E outbreaks occur lack resources and laboratory infrastructure to conduct confirmatory testing of suspected hepatitis E cases. Once a hepatitis E outbreak is suspected, biological samples from suspected cases are shipped to reference laboratories, often in other countries, for confirmation based on detecting HEV RNA using reverse transcriptase polymerase chain reactions (PCR) or IgM antibodies specific to HEV using ELISA. In a displaced persons camp in Niger, for example, health authorities were notified about an increase in acute jaundice syndrome (AJS) cases in January 2017 but “in the absence of appropriate diagnostic capacities” serum samples were sent to Senegal for biological confirmation [6]. Following confirmation of HEV, the outbreak was officially declared in April [6]. The need to wait for confirmation from external laboratories slows down mobilization of resources for public health interventions that can interrupt transmission. These delays also decrease the potential impact of reactive vaccination campaigns, which have the greatest benefits in preventing cases and deaths when implemented in the early stages of an outbreak [7].

Rapid diagnostic tests (RDTs) for hepatitis E, which can detect IgM antibodies or viral antigen [2], require less laboratory infrastructure and can be conducted at point-of-care, but their field performance is not well described. In 2022, the WHO Strategic Advisory Group of Experts on In Vitro Diagnostics (SAGE IVD) conditionally added RDTs for HEV to WHO's list of essential in vitro diagnostics, noting that although additional information on test performance is needed, RDTs can facilitate access to hepatitis E diagnostics in settings where PCR and ELISA are not accessible [8]. Few validation studies have been performed for HEV RDTs and among those that have, they have generally been conducted in controlled laboratory settings with ideal positive and negative samples (eg, samples already known to be positive or negative) and with HEV genotype 3 [9–11]. To our knowledge, no performance data from real world conditions have been published, where antibody or antigen concentrations may vary and where collection and transport conditions can influence test results. Evidence on the field performance of RDTs can help accelerate their broader use in surveillance and outbreak response.

The natural history of HEV, viral, and antibody kinetics and analytical test performance shape the real world performance of HEV diagnostic tests and can challenge our ability to measure their true sensitivity and specificity. Symptoms develop on average 30 days after infection (95% CI 24–36) lasting 1 to 6 weeks [2, 12]. Limited existing evidence suggests that HEV RNA concentrations peak shortly before symptom onset in blood and decay quickly [13], potentially before a case seeks care. After infection, IgM antibodies take time to develop though it is not clear how quickly and how often antibody-based tests may miss early infections [14]. Furthermore, IgM antibodies can last for more than 6 months [15], which can lead to false-positive test results. Assessing the performance of new diagnostic tests without taking into account the imperfections of reference assays and patient characteristics could lead to severe biases in estimates of test performance.

Here, we leverage data from acute jaundice surveillance nested within a vaccine effectiveness study during an outbreak of hepatitis E genotype 1 in South Sudan to generate estimates on the sensitivity and specificity of PCR, IgM ELISA, and IgM RDTs in a real world setting and shed new light on the time course of HEV viremia and antibodies after onset of jaundice.

## METHODS

### Study Setting

Bentiu Internally Displaced Persons camp was established in December 2013 and cases of hepatitis E started to be detected there in 2014 [16]. In March 2022, the South Sudan Ministry of Health in partnership with Médecins Sans Frontières (MSF) implemented the first-ever reactive vaccination campaign with Hecolin [17], coupled with a vaccine effectiveness study. As part of the study, MSF reinforced the use of clinical case definitions and conducted comprehensive testing for all suspected cases [16]. AJS was defined as acute (recent, new, or sudden) onset of yellow coloration of the whites of the eyes or skin, dark urine, or pale clay stools. All cases of AJS were considered suspected cases of hepatitis E.

All suspected hepatitis E cases seeking care at the MSF hospital were identified by clinicians and referred to the study team after consultation or admission. Study staff explained the study objectives and, among suspected cases willing to participate, obtained consent for participation. Adults provided written informed consent while individuals under 18 years of age provided assent and their guardian provided written informed consent. Study staff asked all consenting suspected cases questions about symptoms, date of symptom onset, vaccination status, and sociodemographic variables. To characterize viral and antibody kinetics over time, study participants had a follow-up visit at least 2 weeks after their enrollment where a blood sample was collected.

At each visit, a laboratory technician collected a venous blood sample and prepared all specimens for testing, storage, and transport. Plasma was separated by centrifugation and aliquoted at the MSF hospital, then frozen at −20°C within 6 hours. All samples were transported from Bentiu first to MSF, Juba, South Sudan, then to the reference laboratory in Switzerland on flights with temperature loggers in cold chain using dry ice. Samples were stored in Juba and Geneva at −80°C.

### Laboratory Methods

A trained laboratory technician conducted HEV IgM RDTs and liver function tests at the Bentiu hospital laboratory. The HEV IgM RDT (Assure, Genelabs Diagnostics, Singapore, Republic of Singapore) was performed on ∼35 µL of venous blood. Field staff were trained based on the package insert. The kit was stored at ambient temperature and was performed according to manufacturer's instructions by a trained laboratory technician in Bentiu. AST and ALT concentrations were measured in venous blood using an automated system (Reflotron) or SimplexTAS machine in the hospital laboratory.

PCR and IgM ELISA testing was conducted in a reference laboratory at the University Hospitals of Geneva. We used WANTAI HEV-IgM ELISA and WANTAI HEV-IgG ELISA (WE-7196 and WE-7296, Beijing Wantai Biological Pharmacy Ent.) to detect HEV antibodies in venous plasma following the manufacturer's recommendations.

RNA was extracted from plasma samples using the NucliSens easyMAG instrument (BioMérieux), following the manufacturer's instructions. We used the ampliCube HEV 2.0 Quant real-time quantitative polymerase chain reaction (RT-qPCR) to detect HEV RNA (Mikrogen Diagnostik, Bavaria, Germany) with primers targeting both the HEV ORF2 and ORF3 and built-in internal control for quality assurance [18]. We used a cycle threshold cutoff value of 42 for positivity.

### Statistical Analysis

We first estimated the sensitivity of IgM RDT and IgM ELISA using PCR as the reference standard as a benchmark for how diagnostic evaluations typically assess sensitivity and specificity. We then developed a latent class model to use results from all 3 tests to simultaneously estimate the sensitivity and specificity of each test and account for the imperfect performance of each test [19]. We included each individual's PCR, RDT, and IgM ELISA test results and modeled the combination of the 3 tests as a multinomial likelihood:


$$\begin{array}{rl} & n_{\left(-,-,-\right)},n_{\left(-,-,+\right)},n_{\left(-,+,-\right)},n_{\left(-,+,+\right)},n_{(+,-,-}),n_{\left(+,-,+\right)},n_{\left(+,+,-\right)},\\ & n_{\left(+,+,+\right)}∼\mathrm{multinomial}(\;p_{\left(-,-,-\right)},p_{\left(-,-,+\right)},p_{\left(-,+,-\right)},p_{\left(-,+,+\right)},\\ & p_{\left(+,-,-\right)},p_{\left(+,-,+\right)},p_{\left(+,+,-\right)},p_{\left(+,+,+\right)}) \end{array}$$

The probabilities for each sequence of test results were defined as follows:


$$p(-,-,-)=\;(1-\theta_{1}^{+})(1-\theta_{2}^{+})(1-\theta_{3}^{+})\phi +\theta_{1}^{-}\;\theta_{2}^{-}\;\theta_{3}^{-}\;(1-\phi )$$

$$p(-,-,+)=\;(1-\theta_{1}^{+})(1-\theta_{2}^{+})\;\theta_{3}^{+}\phi +\theta_{1}^{-}\;\theta_{2}^{-}\;(1-\theta_{3}^{-})\;(1-\phi )$$

$$p(-,+,-)=\;(1-\theta_{1}^{+})\;\theta_{2}^{+}\;(1-\theta_{3}^{+})\phi +\theta_{1}^{-}\;(1-\theta_{2}^{-})\;\theta_{3}^{-}\;(1-\phi )$$

$$p(-,+,+)=\;(1-\theta_{1}^{+})\;\theta_{2}^{+}\;\theta_{3}^{+}\;\phi +\theta_{1}^{-}\;(1-\theta_{2}^{-})\;(1-\theta_{3}^{-})\;(1-\phi )$$

$$p(+,-,-)=\;\theta_{1}^{+}\;(1-\theta_{2}^{+})(1-\theta_{3}^{+})\;\phi +(1-\theta_{1}^{-})\;\theta_{2}^{-}\;\theta_{3}^{-}\;(1-\phi )$$

$$p(+,-,+)=\;\theta_{1}^{+}\;(1-\theta_{2}^{+})\;\theta_{3}^{+}\;\phi +(1-\theta_{1}^{-})\;\theta_{2}^{-}\;(1-\theta_{3}^{-})\;(1-\phi )$$

$$p(+,+,-)=\;\theta_{1}^{+}\;\theta_{2}^{+}\;(1-\theta_{3}^{+})\;\phi +(1-\theta_{1}^{-})\;(1-\theta_{2}^{-})\;\theta_{3}^{-}\;(1-\phi )$$

$$p(+,+,+)=\;\theta_{1}^{+}\;\theta_{2}^{+}\;\theta_{3}^{+}\;\phi +(1-\theta_{1}^{-})\;(1-\theta_{2}^{-})\;(1-\theta_{3}^{-})\;(1-\phi )$$

Where *ϕ* denotes the true probability of hepatitis E infection, $\theta^{+}$ denotes test sensitivity, $\theta^{-}$ denotes test specificity, and subscripts 1, 2, and 3 denote PCR, IgM RDT, and IgM ELISA. We included both test results at enrollment and at the follow-up visit in the model and marginalized out the unobserved IgM RDT result at follow-up. Priors and sensitivity analyses using alternate priors are described in Supplementary Tables 5 and 6.

We conducted inference in a Bayesian framework using Hamiltonian Markov Chain Monte Carlo as implemented in the STAN language. In the *unadjusted model*, we treated the sensitivity and specificity of each test as fixed across all individuals. We explored adjusted models where the logit sensitivity of each test varied by days between self-reported jaundice onset and clinic visit (linear or cubic spline with 3 degrees of freedom), age, the interaction between age and days between jaundice onset and clinic visit, and sex (Supplementary Table 3). We explored models with a constant risk of hepatitis E and those that allowed the risk to vary by month of AJS onset. In sensitivity analyses, we explored the use of models that relaxed the assumption of conditional independence between test results by including a patient-level random effect when estimating logit sensitivity and logit specificity [19].

For each model, we ran 4 chains of 1000 iterations after warm-up and assessed convergence among chains visually and using the Gelman-Rubin R-hat statistic [20]. We used approximate leave-one-out cross-validation using Pareto smoothed importance sampling [21] to compare the predictive accuracy of each model and select the final model.

We used accelerated failure time models to obtain parametric and nonparametric estimates—accounting for the censored nature of the data—of the median time from jaundice onset to first negative test result among individuals who tested positive at enrollment, for PCR and IgM ELISA separately [22]. We explored Weibull, log-normal, and gamma distributions for the survival curve and selected the distribution that minimized the Akaike Information Criterion (AIC). We quantified uncertainty using the 2.5th and 97.5th percentiles of 1000 bootstrap draws. In sensitivity analyses, we explored whether the time to first negative test varied by age (linear and categorical) and/or sex.

We conducted posterior retrodictive checks for the accelerated failure time models to assess how well the final selected models could reproduce observed diagnostic test results. Code for all analyses and a minimal dataset are available at https://github.com/HopkinsIDD/hev-diagnostics-bentiu.

### Ethics

Ethical approval for the parent study was obtained from MSF (ERB #2167), the South Sudan Ministry of Health Research Ethics Board (RERB-MOH # 54/27/09/2022) and The Johns Hopkins Bloomberg School of Public Health Institutional Review Board (IRB00025966).

## RESULTS

We identified 893 suspected hepatitis E cases between March and December 2022 with complete test results at enrollment (Table 1). Of these, 67% (*N* = 602) had jaundice onset within 1 week and 90% (*N* = 805) within 30 days of their clinic visit. Among suspected hepatitis E cases, 180 (20%) had viral RNA detected by PCR, 245 (27%) had IgM antibodies detected by RDT, and 236 (26%) had IgM antibodies detected by ELISA. Half of suspected cases were female (46%) and half were under 16 years old (49%). A higher proportion of suspected cases who tested positive on all tests were under 16 years old compared to suspected cases who had no viral RNA or IgM antibodies detected (67% vs 42%). 24 suspected cases were pregnant, of whom 23 were pan-negative and 1 was RDT-positive only. A majority of suspected cases had IgG antibodies detected by ELISA at the time of enrollment (85%). A higher proportion of pan-positive suspected cases returned for a follow-up visit with a blood draw compared to mixed and pan-negative suspected cases (46% vs 40% vs 39%). We excluded 1 suspected case missing jaundice onset date (0.11%) from subsequent analyses.

**Table 1.** Characteristics of Suspected Hepatitis E Cases Who Presented to Care at a Health Facility Between March and December 2022 With Complete Testing Data

	Case Type At Enrollment			
Characteristic *N* (col %)	Overall *N* = 893	Pan-positive *N* = 169	Mixed *N* = 107	Pan-negative *N* = 617
Female	406 (45.5)	77 (45.6)	48 (44.9)	281 (45.5)
Age, median (IQR)	15.5 (5.9, 26.2)	9.6 (4.9, 17.7)	13.3 (5.4, 21.5)	18.3 (6.9, 28.5)
Age				
0–5	192 (21.5)	44 (26.0)	26 (24.3)	122 (19.8)
6–15	241 (27.0)	69 (40.8)	34 (31.8)	138 (22.4)
16–39	379 (42.4)	50 (29.6)	38 (35.5)	291 (47.2)
40+	81 (9.1)	6 (3.6)	9 (8.4)	66 (10.7)
Days since jaundice onset^a^, median (IQR)	5.0 (3.0, 10.0)	4.0 (3.0, 7.0)	5.0 (3.0, 15.0)	5.0 (3.0, 12.0)
Days since jaundice onset^a^				
≤1 week	602 (67.4)	132 (78.1)	64 (59.8)	406 (65.9)
>1 to 2 weeks	129 (14.4)	30 (17.8)	15 (14.0)	84 (13.6)
>2 weeks to 1 month	74 (8.3)	6 (3.6)	10 (9.3)	58 (9.4)
>1 to 2 months	49 (5.5)	1 (0.6)	9 (8.4)	39 (6.3)
>2 months	38 (4.3)	0 (0)	9 (8.4)	29 (4.7)
Anti-HEV IgG positive	763 (85.4)	169 (100)	97 (90.7)	497 (80.6)
Elevated ALT^b^	135 (20.5)	98 (72.1)	4 (5.2)	33 (7.4)
Elevated AST^c^	55 (17.5)	8 (25.0)	4 (10.3)	43 (17.6)
Hospitalized^d^	57 (6.4)	6 (3.6)	13 (12.1)	38 (6.2)
Died	9 (1.0)	0 (0)	2 (1.9)	7 (1.1)
Attended follow-up visit	363 (40.6)	77 (45.6)	43 (40.2)	243 (39.4)

Abbreviations: Pan-positive, PCR, IgM ELISA, and IgM RDT+; Pan-negative, PCR, IgM ELISA and IgM RDT−.

^a^Missing for 1 suspected case.

^b^Missing for 233 suspected cases. For males, ALT ≥41 units per liter was considered elevated. For females, ALT >32 units per liter was considered elevated [23, 24].

^c^Missing for 578 suspected cases. AST above 40 units per liter was considered elevated.

^d^Missing for 1 suspected case.

### Diagnostic Performance

Using PCR as the reference standard, the sensitivity of IgM RDT was 94.4% (95% CI: 90.0, 97.3) and specificity was 89.5% (95% CI: 87.0, 91.6) (Table 2). The sensitivity and specificity of IgM RDT compared to IgM ELISA was 88·1% (95% CI 83.3, 92.0) and 94.4% (95% CI: 92.3, 96.0), respectively.

**Table 2.** Sensitivity and Specificity of Rapid Diagnostic Tests (RDT) Compared to Reverse Transcription Polymerase Chain Reaction (PCR) and Enzyme-linked Immunosorbent Assays (ELISA)

	Gold Standard		Metric (95% CI)	
Test results	PCR+	PCR−	Sensitivity	Specificity
IgM RDT+	170	75	94.4% (90.0, 97.3)	89.5% (87.0, 91.6)
IgM RDT−	10	637		
	IgM ELISA+	IgM ELISA−	Sensitivity	Specificity
IgM RDT+	208	37	88.1% (83.3, 92.0)	94.4% (92.3, 96.0)
IgM RDT−	28	619		

**Figure 1.**
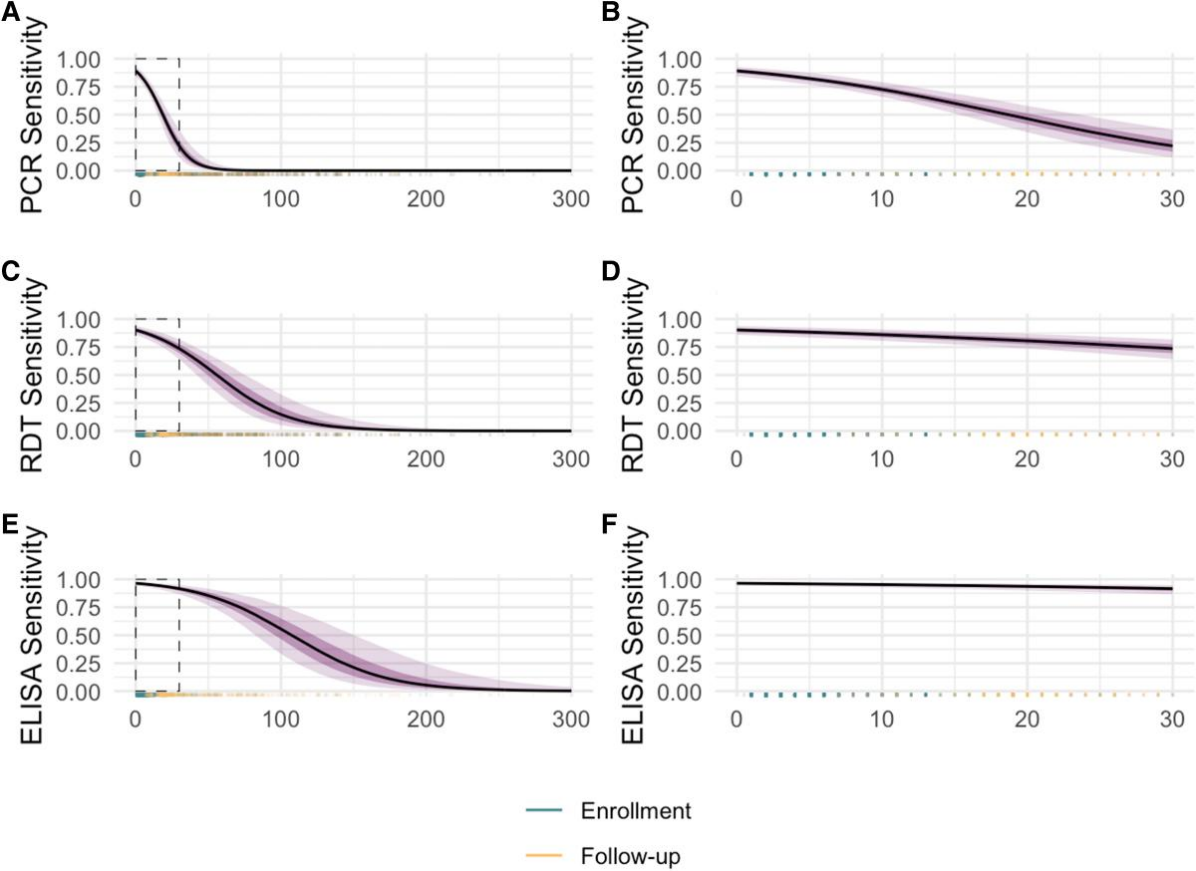
Sensitivity and specificity of diagnostic tests for detecting hepatitis E infections as a function of days between jaundice onset and clinic visit. (*A*) PCR sensitivity across all care-seeking delays with box around the first 30 days between jaundice onset and clinic visit; (*B*) PCR sensitivity in the first 30 days between jaundice onset and clinic visit; (*C*) IgM RDT sensitivity across all care-seeking delays with box around the first 30 days between jaundice onset and clinic visit; (*D*) IgM RDT in the first 30 days between jaundice onset and clinic visit; (*E*) IgM ELISA sensitivity across all care-seeking delays with box around the first 30 days between jaundice onset and clinic visit; (*F*) IgM ELISA in the first 30 days between jaundice onset and clinic visit; Rug plot showing frequency of observed values. Line indicates median, darker shading around line indicates 20th and 80th quantiles, and lighter shading indicates 2.5th and 97.5th quantiles. Average sensitivity and specificity by test with 95% credible interval. Logit sensitivity varied linearly by days between self-reported jaundice onset and clinic visit, separately for each test.

When using a latent class model that considers all 3 HEV-specific test results as imperfect, we estimated that 24.8% (95% CI: 22.2, 27.6) of suspected cases were true HEV infections. With this latent class model, we estimate the average specificity of PCR was 98.2% (95% Credible Interval [CrI]: 97.6, 98.7), IgM RDT was 94.7% (95% CrI: 93.4, 95.9), and IgM ELISA to be 94.6% (95% CrI: 93.0, 95.9) (Table 3). Models allowing for sensitivity to decrease with increasing care-seeking delays were well supported by the data (Supplementary Table 3). When averaged across observed care-seeking delays up to 30 days, PCR had the lowest sensitivity (73.4%, 95% CrI 27.2, 90.0), followed by IgM RDT (86.2%, 95% CrI: 74.0, 92.5) and IgM ELISA (95.2%, 95% CrI: 90.9, 97.7) (Table 3). The median sensitivity of PCR was 89.2% among individuals presenting for care the day of jaundice onset (95% CrI: 84.2, 92.7), 60.1% after 2 weeks (95% CrI 51.0, 68.8), and 20.2% after 1 month (95% CrI: 10.4, 34.7) (Figure 1). The sensitivity of RDT was 90.2% among those presenting for care the day of jaundice onset (95% CrI: 85.9, 93.8), 83.4% after 2 weeks (95% CrI: 77.3, 88.8), and 72.7% after 1 month (95% CrI: 62.8, 81.3). The sensitivity of IgM ELISA was 96.5% among those presenting for care the same day as jaundice onset (95% CrI: 94.1, 97.9), 94.5% after 2 weeks (95% CrI: 91.3, 96.7) and 91.4% after 1 month (95% CrI: 86.3, 94.7). In models that allowed the decline in sensitivity by care-seeking delays to vary by age group, the sensitivity of all diagnostic tests, most notably those based on IgM, declined more quickly for individuals under 5 years of age compared to over 5 years (Supplementary Figure 6). Alternative models, including those with different definitions for symptom onset, different priors, and conditional dependence structure led to similar estimates of test performance (Supplementary Tables 3 and 6).

**Table 3.** Sensitivity and Specificity of Diagnostic Tests for Detecting True Hepatitis E Infections based on Latent Class Analysis

	Performance Metric (95% Credible Interval)		
Test	Sensitivity: Care seeking within 14 d of AJS onset	Sensitivity: Care seeking within 30 d of AJS onset	Specificity
PCR	82.0% (64.8, 90.3)	73.4% (27.2, 90.0)	98.2% (97.6, 98.7)
IgM RDT	88.2% (82.3, 92.6)	86.2% (74.0, 92.5)	94.7% (93.4, 95.9)
IgM ELISA	95.8% (93.1, 97.8)	95.2% (90.9, 97.7)	94.6% (93.0, 95.8)

Abbreviation: AJS, acute jaundice syndrome.

Among suspected cases with care-seeking delays up to 30 days the average positive predictive value (PPV) for PCR was 85.7% (95% CI: 37.0, 98.1), IgM RDT was 75.2% (95% CI: 20.2, 95.1), and IgM ELISA was 76.2% (95% CI: 21.3, 95.4). The average negative predictive value (NPV) among suspected cases with care-seeking delays up to 30 days for PCR was 91.6% (95% CI: 67.0, 99.7), for IgM RDT was 95.0% (95% CI: 83.5, 99.8), and for IgM ELISA was 98.2% (95% CI: 93.5, 99.9).

As PPV and NPV are influenced by care-seeking delays (through impacting sensitivity) and the underlying prevalence of hepatitis E, we explored different hypothetical scenarios to demonstrate how test interpretation may differ in other settings. As prevalence increased, the PPV of all tests was higher and with less differences between tests (Supplementary Figure 2). Increasing care-seeking delays and prevalence reduced the NPV of all tests but had the strongest effect on the NPV of PCR.

### Time Course of HEV Viremia and IgM Antibodies

In the subset of participants who returned for a follow-up visit with a blood draw (*N* = 363), samples at enrollment were collected a median of 4 days after jaundice onset (IQR: 3–9; range: 0–217) while follow-up samples were collected a median of 36 days after jaundice onset (IQR: 21,77; range: 7–470) (Supplementary Table 7).

**Figure 2.**
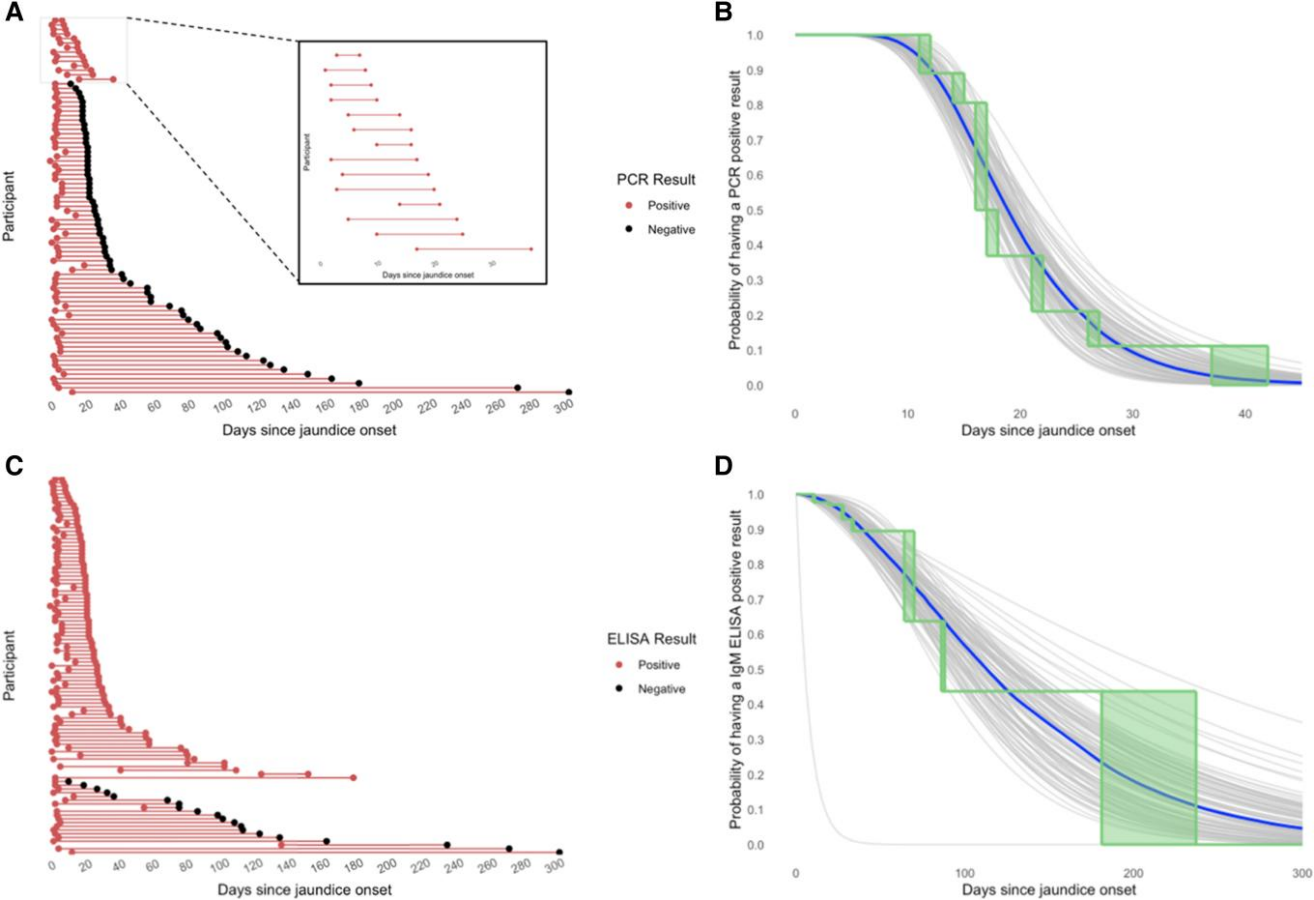
Longitudinal PCR and IgM ELISA results among suspected hepatitis E cases with a positive PCR and/or IgM ELISA result at enrollment. (*A*) Cohort plot of participants who were PCR positive at enrollment with a follow-up test result. Each entry on the y-axis represents 1 participant. (*B*) Parametric and nonparametric estimates of the probability of viremia at follow-up among participants who were viremic at enrollment based on serum PCR by days since jaundice onset. The best fitting parametric model assumed a log-normal distribution. (*C*) Cohort plot of participants who were IgM ELISA positive at enrollment with a follow-up test result. (*D*) Parametric and nonparametric estimates of the probability of IgM seropositivity at follow-up among participants who were seropositive based on serum ELISA at enrollment by days since jaundice onset. The best fitting parametric model assumed a gamma distribution. Rectangular regions in panel (*B*) and (*D*) represent estimates with similar likelihood in the nonparametric survival curve. Lighter curves represent bootstrapped survival probability curves, bolded curve represents median of all bootstrapped curves. Excludes 4 suspected cases who had indeterminate IgM ELISA results at follow-up.

Among suspected cases who were PCR-positive at enrollment (*N* = 83) only 14 (17%) were positive at follow-up (Figure 2), with a median time from jaundice onset to having a negative PCR result of 18.9 days (95% CI: 16.9, 21.3). Times to a negative PCR-result did not vary significantly by age or sex. Among suspected cases who were IgM ELISA-positive at enrollment (*N* = 104), 81 (78%) were still positive at follow-up, with a median time from jaundice onset to a negative IgM ELISA of 113.0 days (95% CI: 87.4, 163.1). Time to a negative IgM ELISA did not vary by sex but individuals under 5 years old had a faster time to a negative IgM ELISA test (median: 61.6 days; 95% CI: 39.4, 90.2) compared to individuals over 5 (median: 125.8 days; 95% CI: 93.1, 184.5) (Supplementary Figure 7).

## DISCUSSION

Diagnostic tests for hepatitis E had high sensitivity and specificity in field conditions, though sensitivity decreased with delayed care-seeking postjaundice onset. The assure RDT demonstrated higher sensitivity than PCR and similar specificity to ELISA, underscoring its potential utility in surveillance for understanding burden of disease and detecting outbreaks. Negative PCR tests may not rule out current hepatitis E, particularly among individuals with modest delays between jaundice onset and care seeking. Our findings provide a pathway for strengthening diagnostic capacities for identifying hepatitis E cases in settings that are most likely to have limited laboratory infrastructure and be most impacted by hepatitis E genotypes 1 and 2.

Our crude estimates of IgM RDT sensitivity compared to PCR and/or IgM ELISA are similar to existing published literature from laboratory studies [11]. Our estimates of PPV are not generalizable to other settings with different underlying hepatitis E prevalence and care-seeking behavior, but our results can be used by decision-makers to generate setting-specific ranges of plausible PPVs. Though PCR/ELISA confirmation may still be necessary for official outbreak declarations, RDT results could help expedite outbreak response activities and help interrupt hepatitis E transmission in the critical early days of outbreaks.

Our adjusted estimates of the average sensitivity and specificity of each diagnostic test are based on the distribution of days between jaundice onset and care seeking in our study population. The proportion of suspected cases in our study with jaundice onset within 2 weeks of clinic visit at enrollment (82%) is notably higher than among hospitalized patients in an acute jaundice surveillance study in Bangladesh (39%) [23]. Differences in care seeking could be due to the potentially shorter distance needed to travel to the MSF hospital within Bentiu IDP camp or due to increased care seeking given awareness about the ongoing HEV outbreak. Care-seeking delays in our study population are similar to observed delays during a hepatitis E outbreak in a displaced persons camp in Darfur, Sudan (5 days) [24], suggesting care-seeking delays may be shorter in camp settings with free healthcare compared to non-camp settings. Notably, the PPV of RDTs did not vary greatly across different hypothetical delays in care seeking (Supplementary Figure 2).

We found faster IgM seroreversion in children under 5 compared to others, and some evidence HEV RNA clearance may also be faster in children under 5, though to a lesser extent. For some viruses, young children do not mount robust antibody responses upon primary exposure and have lower peak antibody titers relative to older individuals [25]. Lower peak antibody concentrations in young children, even if IgM decay rates were similar to adults, would lead to quicker IgM seroreversion. Further stratifying children in our study population illustrates that children under 4 years old had faster IgM decay compared to children 4 to 5 years (Supplementary Figure 8), suggesting that the youngest children are likely driving this trend. Interestingly, studies from Bangladesh have shown more frequent seroreversion of anti-HEV IgG antibodies in young children [26, 27]. Additional research tracking quantitative measures of IgM antibodies can help better explain the apparent age-related differences in kinetics.

Our analyses have a number of limitations. Jaundice onset date in our study was self-reported, and jaundice can be difficult to identify in general and particularly in populations with darker skin [28, 29]. If self-reported jaundice onset date is systematically reported to be later than true date, we would underestimate the number of days between jaundice onset and care seeking. We expect this type of misclassification to have occurred nondifferentially among HEV infected and uninfected suspected hepatitis E cases. Importantly, our results are based only on cases seeking care at the hospital (inpatient and outpatient). Our estimates for the sensitivity of each diagnostic test could be overestimates if cases with milder symptoms, lower HEV viral load, and/or lower IgM response were less likely to seek care. Our sensitivity and specificity estimates may not be generalizable to other settings due to Bentiu IDP camp's history of repeated hepatitis E outbreaks. Infection with HEV generates long-lasting IgG antibodies, which could influence the sensitivity of diagnostic tests in subsequent infections. IgG positivity can reflect antibodies from a previous and/or current HEV infection, or vaccination. Animals previously exposed to HEV with high avidity anti-HEV IgG antibodies had shorter viremia, lower HEV RNA levels, and no detectable IgM antibody response compared to animals with lower avidity IgG when reinfected with HEV genotype 1 [30]. We used venous blood for RDTs as opposed to capillary blood, which could impact RDT performance if venous blood has different fluid dynamics in lateral flow chromatographic assays and depending on staff training and comfort performing each procedure according to manufacturer's instructions. Vaccination against hepatitis E could have influenced the time course of RNA or IgM antibodies in vaccinated individuals with breakthrough infections, but only 36 individuals with detectable RNA or IgM antibodies at enrollment reported being vaccinated. Less than half of suspected cases returned for a follow-up visit. Hepatitis E severity may be associated antibody response, therefore if individuals who returned for testing were less likely to have been severe cases; our estimates of median time from cases’ first ELISA positive result to having a negative result may be underestimated.

We provide new insights on both the time-varying sensitivity of IgM RDTs and the traditional “gold-standard” assays, which can help inform test interpretation and future surveillance guidance. Strengthening investments in surveillance in settings where hepatitis E transmission occurs can help us more rapidly detect and respond to outbreaks and develop more data-informed strategies to using hepatitis E vaccines both in endemic and epidemic settings.

## Supplementary Material

jiaf436_Supplementary_Data

## Supplementary Data

Supplementary materials are available at *The Journal of Infectious Diseases* online (http://jid.oxfordjournals.org/). Supplementary materials consist of data provided by the author that are published to benefit the reader. The posted materials are not copyedited. The contents of all supplementary data are the sole responsibility of the authors. Questions or messages regarding errors should be addressed to the author.
